# PRESIDENTIAL SYMPOSIUM: EMBRACING OUR DIVERSITY IN GERONTOLOGY EDUCATION: PERSPECTIVES ON STRATEGIES AND LEARNERS

**DOI:** 10.1093/geroni/igac059.863

**Published:** 2022-12-20

**Authors:** Joann Montepare, Dana Bradley, Tamara Baker

## Abstract

Our populations are not only becoming older, but also becoming more diverse on several fronts. Our new social structures call for new strategies for embracing our diversity in how we design and deliver education about aging in and beyond the classroom. In this AGHE Presidential Symposium, educators will discuss innovative and needed ways we can integrate diversity in gerontological pedagogical practices. To begin, Rona Karasik (St. Cloud State University) will discuss the call for integrating anti-racist strategies in classroom practices, and offer examples of several such strategies. Next, Brian Carpenter (Washington University in St. Louis) will discuss shrinking geropsychology pipelines, especially for students from racial and ethnic groups, and will share recommendations for expanding these pipelines. Aaron Guest (Arizona State University) will then discuss the need to extend diversity and education efforts to include LGBTQ individuals and issues. Karen Lincoln (University of Southern California) will discuss the need to move educational efforts beyond our classrooms to diverse learners in our communities. To this end, she will describe a dynamic academic−community partnership that provides aging-focused education to African-American older adults. Dana Burr-Bradley (University of Maryland, Baltimore County) will give the final presentation with a discussion about the need for an international lens that appreciates the global diversity of the aging experience. Tamara Baker (University of North Carolina, Chapel Hill) will serve as the discussant and offer her perspective on how the need to embrace our diversity in gerontology education cuts across all GSA units and connects them with AGHE’s educational mission.

